# Detection of African Swine Fever Virus in Feed and Feed Mill Environment Following Extended Storage

**DOI:** 10.1155/2023/3455128

**Published:** 2023-08-23

**Authors:** Grace E. Houston, Jessie D. Trujillo, Cassandra K. Jones, Taeyong Kwon, Charles R. Stark, Konner Cool, Chad B. Paulk, Natasha N. Gaudreault, Jason C. Woodworth, Igor Morozov, Carmina Gallardo, Jordan T. Gebhardt, Jürgen A. Richt

**Affiliations:** ^1^Department of Diagnostic Medicine/Pathobiology, Kansas State University, Manhattan, KS, USA; ^2^Center of Excellence for Emerging and Zoonotic Animal Disease, Kansas State University, Manhattan, KS, USA; ^3^Department of Animal Science and Industry, College of Agriculture, Kansas State University, Manhattan, KS, USA; ^4^Department of Grain Science and Industry, College of Agriculture, Kansas State University, Manhattan, KS, USA; ^5^Instituto Nacional de Investigación y Technología Agraria y Alimentaria, Animal Health Research Centre, Madrid, Spain

## Abstract

One way to mitigate risk of feed-based pathogens for swine diets is to quarantine feed ingredients before inclusion in complete diets. Data have been generated evaluating the stability of swine viruses in ingredients, but the stability of African swine fever virus (ASFV) in feed or in a feed manufacturing environment has not been well characterized. Therefore, this study aimed to determine the stability of ASFV DNA in swine feed and on mill surfaces over time. A pilot-scale feed mill was used to manufacture six sequential batches of feed consisting of a batch of ASFV-free feed, followed by a batch inoculated with ASFV (final concentration = 5.6 × 10^4^ TCID_50_/g), and then four subsequent ASFV-free batches. After each batch, 10 feed samples were aseptically collected in a double “X” pattern. During feed manufacturing, 24 steel coupons were placed on the floor of the manufacturing area and allowed to collect dust during feed manufacturing. Once feed manufacturing was completed, feed samples and steel coupons were stored at room temperature. Three of each were randomly selected from storage on 3, 7, 14, 28, 60, 90, and 180 days after feed manufacturing and analyzed for ASFV DNA. For feed samples, there was evidence of a batch × day interaction (*P* = 0.023) for the quantification of genomic copies/g of feed, indicating that the amount of ASFV DNA present was impacted by both the batch of feed and days held at room temperature. There were no differences of genomic copies/g in early batches, but quantity of detectable ASFV decreased with increasing storage time. In Batches 4–6, the greatest quantity of ASFV DNA was detected on the day of feed manufacturing. The lowest quantity was detected on Day 7 for Batch 4, Day 60 for Batch 5, and at 28 and 180 days for Batch 6. There was no evidence of ASFV degradation on environmental discs across holding times (*P* = 0.433). In conclusion, the quarantining of feed may help reduce but not eliminate the presence of ASFV DNA in feed over time. Importantly, ASFV DNA was detectable on feed manufacturing surfaces for at least 180 days with no overt evidence of reduction, highlighting the importance of bioexclusion of ASFV within feed manufacturing facilities and the need for thorough/effective decontamination and other mitigation processes in affected areas.

## 1. Introduction

African swine fever virus (ASFV) can have devastating agricultural and economic consequences when introduced to a region which puts the United States' swine industry at risk as it maintains trade with ASFV endemic countries for feed ingredients [1]. Recent data suggest certain feed ingredients have the ability to support virus survival during simulated conditions of transatlantic shipping [2, 3]. To prevent the spread of disease, feed manufacturing facilities hold, or quarantine, these ingredients before including them in complete swine diets [2]. Periods of quarantine allow for viruses to naturally decay, thereby reducing infectivity (viral loads) within the feed over time [4, 5]. However, this practice of extended storage of complete feed may result in quality concerns because of ingredient instability over time, although little data are available regarding vitamin stability in complete feed during the extended storage. When considering research that has evaluated ASFV persistence, there is unanimous agreement that ASFV has the ability to persist for long periods of time in cured meats [6], fat sources [7], soil and wild boar carcasses [8], and feces, urine, and oral fluids [9]. However, when trying to extrapolate these findings to feed ingredients and feed manufacturing facilities, it can be challenging to define the limit to which ASFV is affected since most of this research has focused on porcine epidemic diarrhea virus (PEDV), an enveloped RNA virus. How these studies apply to ASFV, a DNA virus with proven environmental stability, remains uncertain. Therefore, the objectives of this study were (i) to evaluate the impact of storage over a period of 6 months at room temperature of ASFV-contaminated complete swine feed on ASFV stability and (ii) to evaluate the length of time that ASFV could be detected in a feed mill environment after its introduction through the milling of experimentally ASFV-inoculated swine feed.

## 2. Materials and Methods

### 2.1. General

This research has been reported as part of the doctoral dissertation of the first author Houston [10]. Neither humans nor animals were used as research subjects in this experiment, so relevant approvals were not applicable. The study was conducted at the Biosecurity Research Institute (BRI) in Manhattan, KS, with approval by the Kansas State University Institutional Biosafety Committee (project approval #1427.1). The feed manufacturing process was done within a biosafety level- (BSL-) 3Ag large animal room while laboratory work was done within a BSL-3+ laboratory space.

### 2.2. Inoculation and Sampling

Eight and a half millilitres of pooled blood treated with ethylendiaminetetraacetic acid (EDTA) from ASFV infected pigs was mixed in RPMI media to prepare 530 mL of the virus inoculum at the final concentration of 2.7 × 10^6^ TCID_50_/mL of ASFV genotype II virus (Armenia 2007). Feed was manufactured as described by Elijah et al. [11] and Elijah et al. [12]. In brief, the feed manufacturing system was first primed with an ASFV-free batch of feed which was subsequently followed by a second batch of feed that was contaminated with ASFV. Four additional batches of ASFV-free feed were then mixed and discharged through the same equipment without any cleaning or disinfection occurring between batches. For this study, a corn and soybean-meal based diet with a composition normally fed to gestating sows was manufactured at the Kansas State University O.H. Kruse Food Technology Innovation Center (Manhattan, KS) and transported to the BSL-3 facility. Feed manufacturing was structured as follows:

Negative Control (Batch 1)—Priming the feed mill: to initiate the trial, a 25 kg batch of ASFV-free feed was mixed in a 0.113 m^3^ electric paddle mixer (H.C Davis Sons Manufacturing, model # SS-L1; Bonner Springs, KS). The feed was mixed for 5 min then discharged at a rate of approximately 4.5 kg/min into the conveyor (Universal Industries, Cedar Falls, IA) that carried 74 buckets (each 114 cm^3^) of feed. The feed was conveyed and discharged through a downspout into double-lined bags.

Positive Control (Batch 2)—ASFV-contaminated feed: upon completion of priming the system with the initial batch of ASFV-free feed, 530 mL of a genotype II (Armenia 2007) ASFV (2.7 × 10^6^ TCID_50_/mL) was then mixed with 4.7 kg of feed in a 5 kg stainless steel mixer (Cabela's Inc., Sidney, NE) to make 5.23 kg (5.6 × 10^4^ TCID_50_/g) of ASFV-contaminated feed which was subsequently added to 20 kg of feed and then mixed, conveyed, and discharged using the same equipment and procedures as previously described for the negative control.

Sequences 1–4 (Batches 3, 4, 5, and 6)—Milling of subsequent batches of feed: following discharge of the positive control batch of feed, the same process of mixing, conveying, and discharging 25 kg batches of feed was repeated four additional times using ASFV-free feed.

After a batch of feed was discharged, 10 feed samples were collected using methods described by Jones et al. [13]. In brief, 10 samples were collected from the feed that had been discharged in a biohazard tote by making two “X” patterns. This sampling pattern was done eight separate times after every batch to accumulate the appropriate number of samples used to assess ASFV DNA quantity/stability at Day 0 and after 3, 7, 14, 28, 60, 90, and 180 days of holding time in room temperature (RT) storage. Once feed manufacturing was completed, all feed samples were transported to a BSL-3+ laboratory for ASFV DNA analysis. Day 0 manufacturing feed samples were analyzed as previously described and included in Elijah et al. [11, 12] as part of the data analysis. All other feed samples were stored at RT for the intended storage time. On 3, 7, 14, 28, 60, 90, and 180 days after feed manufacturing, the 10 corresponding feed samples for each batch were removed from RT storage and 3 feed samples were randomly selected for ASFV DNA analysis.

Nine stainless steel coupons (Built So-Well Manufacturing, Manhattan, KS), 10 × 10 cm in size, referred to as environmental discs, were placed at floor level in three different locations within the BSL-3 Ag large animal room (one location near the feed manufacturing equipment and two locations in different corners of the room, outside the working area). Environmental discs (*n* = 27) were allowed to collect dust during the feed manufacturing process and rested overnight. The following day all environmental discs were placed into a storage container and stored at RT in a locked cabinet. At Days 0, and 3, 7, 14, 28, 60, 90, and 180 days after feed manufacturing, one environmental disc from each of the three locations were randomly selected and sampled (*n* = 3) using a 10 × 10 cm cotton gauze as previously described [11]. Remaining environmental discs not used for this analysis were discarded at the conclusion of the study following BSL-3 laboratory protocols for disposal.

### 2.3. Laboratory Analysis

Feed samples and environmental swabs from environmental discs were processed and tested in a BSL-3 laboratory in the BRI. For the feed samples, each 10 g sample was put in a 50 mL conical tube, suspended in 35 mL of PBS, the tube capped and inverted, then incubated overnight at 4°C. Approximately 10 mL of supernatant was recovered, aliquoted into 5 ml cryovials, and stored at −80°C until processed for qPCR. For environmental swabs from environmental discs, 20 mL of phosphate buffered solution was added to each swab within a 50 ml conical tube, tube capped, inverted, and incubated overnight in 4°C. Tubes were vortexed for about 30 s and held upright for 5 min. Approximately 10 mL of supernatant was recovered, aliquoted into 5 mL cryovials, and stored at −80°C until processed for qPCR.

Feed samples and environmental disc samples were processed in a similar manner for the recovery of DNA for PCR testing. In preparation for magnetic bead-based DNA extraction, 500 *µ*L of PBS eluent was combined with 500 *µ*L of Buffer AL (Qiagen, Germantown, MD, USA), briefly vortexed, and incubated at 70°C for 10 min in an oscillating heat block. DNA extraction was carried out using the GeneReach DNA/RNA extraction kit on a Taco™ mini automatic nucleic acid extraction system (GeneReach, Boston, MA, USA). The extraction was performed according to the manufacturer's instructions with modifications. In brief, 200 *µ*L of AL sample lysate was transferred to Column A of the taco deep-well extraction plate which contained 500 *µ*L of the GeneReach lysis buffer and 50 *µ*L of magnetic beads and mixed by pipetting. Two hundred microlitres of molecular grade isopropanol (ThermoFisher Scientific, Waltham, MA, USA) was added to this well prior to extraction. The extraction consisted of two washes with 750 *µ*L of wash Buffer A, one wash with 750 *µ*L wash Buffer B, and a final wash with 750 *µ*L of 200 proof molecular grade ethanol (ThermoFisher Scientific). After a 5 min drying time, DNA was eluted with 100 *µ*L elution buffer and subsequently transferred into 1.5 mL DNA/RNA-free centrifuge tubes (VWR) for storage. Positive and negative extraction controls were included in sample processing and consist of the positive extraction control, a plasmid containing partial sequence of the ASFV p72 and PCR-grade water (negative).

Real-time quantitative PCR (qPCR) was carried out using primers and probes designed to detect the gene encoding for ASFV p72 and PerfeCTa® FastMix II® (Quanta Biosciences, Gaithersburg, MD, USA) on the CFX96 Touch™ Real-Time PCR Detection System (Bio-Rad, Hercules, CA, USA). The qPCR reactions were performed in duplicate with each well containing 5 *µ*L of DNA, 0.2 *µ*L (200 nM) of each primer (Integrated DNA Technology, Coralville, IA, USA), and 0.4 *µ*L (200 nM) of FAM probe (Thermo Fisher Scientific) in a total reaction volume of 20 *µ*L. Thermocycling conditions were 95°C for 5 min, followed by 45 cycles of 95°C for 10 s and 60°C for 1 min.

ASFV p72 genomic copy number (CN) was calculated using reference standard curve methodology using a 10-point reference standard curve composed from tenfold serial dilutions performed in triplicate of a quantitated ASFV p72 plasmid DNA control. CN for samples were mathematically determined using the PCR-determined mean cycle threshold (Ct) for ASFV p72 (two PCR well replicates) and the slope and intercept of the ASFV p72 DNA standard curve. Data are reported as PCR determined copy number per mL. Genomic CN/g for each sample was based upon the genomic CN/mL of solution recovered during sample processing, multiplied by the volume of phosphate buffered solution added during sample processing (35 mL), then divided by the amount of feed per suspension (10 g).

### 2.4. Statistical Analysis

Statistical analysis was performed using R programing language (Version 3.6.1 (2019-07-05), R Core Team, R Foundation for Statistical Computing, Vienna, Austria). Experimental units were the feed and environmental samples. Each feed and environmental sample had one extraction for PCR assay and each extraction was run in duplicate for PCR analysis. However, for feed sample results from Batch 2 on Day 1, each feed sample had two extractions for PCR assay and both extractions were run in duplicate for the PCR analysis.

For feed samples, response values for the ASFV P72 gene were analyzed using a linear model fit with the lme function in the nlme packing and a normal distribution with the fixed effect as batch, day, and the associated interaction with a random effect of sample to indicate the appropriate level of experimental replication given the duplicate qPCR analysis of feed samples.

For environmental discs, response values for the ASFV P72 gene were analyzed using a linear model fit with the lme function in the nlme packing and a normal distribution with the fixed effect as day with a random effect of sample.

Results of Ct and quantification of genomic copies are reported as least square means ± standard error of the mean. Samples not containing detectable ASFV DNA were assigned a value of 45 because that was the greatest number of cycles the qPCR assay was performed before concluding a sample did not have detectable ASFV DNA. Genomic copy data were transformed with log_10_ function and analysis included PCR negative reactions using a value of 0 for the quantified genomic CN/mL or CN/g. All statistical models were evaluated using visual assessment of studentized residuals and models accounting for heterogeneous residual variance were used when appropriate. A Tukey multiple comparison adjustment was incorporated into all statistical models. Results were considered significant at *P* ≤ 0.05 and marginally significant between *P* > 0.05 and *P* ≤ 0.10.

## 3. Results and Discussion

ASFV is the only member of the viral family *Asfaviridae* with *Ornithodoros* ticks serving as biological vectors and reservoir hosts besides wild boars, wart hogs, and other wild suid species [14]. Transmission of ASFV has been documented via direct contact with infected pigs or indirect contact through contaminated fomites. Additionally, ASFV can be detected in various feed matrices subjected to transatlantic shipping conditions and has been shown to be highly stable within the environment in a feed production system, specifically in areas of high-foot traffic and on worker's clothing [1–4, 15, 16]. An understanding of ASFV properties in swine feed and a feed mill environment is pivotal since the US relies on trade with ASFV endemic countries for feed ingredients for the swine feed [17]. While these feed ingredients are manufactured in settings that are regulated and controlled by third party auditors, there is still the potential that these feed ingredients could become contaminated with ASFV during shipping and transportation. Due to the necessity of this trade relationship, research has focused on understanding the implications of ASFV contamination into a feed mill, how feed batch sequencing helps to reduce contamination in the subsequent batches and how this impacts the feed mill environment [11, 12]. This work is critical to prevent further spread of ASFV, which has expanded to regions of Asia and Europe and confirmed to be present in the western hemisphere (Hispaniola island) for the first time in 40 years [14, 18]. However, there are gaps in understanding the impacts of holding times on ASFV contaminated feed and how long ASFV can persist in a feed mill environment. When considering previous research, it has been documented that feed mills could harbor PEDV in the feed mill environment for long periods of time and decontamination of the feed mill would be largely impractical if contaminated with PEDV [19, 20]. When comparing the two viruses, it could be assumed that ASFV could persist within a feed mill environment and feed for longer periods of time due to innate qualities of this virus but there is limited research on this topic at this time. Therefore, this study sought to understand how sequencing batches of feed impacted quantity of ASFV in swine feed over various holding times and how long ASFV can be detected in environmental samples collected from feed manufacturing surfaces.

Batch 1 feed samples were PCR negative, as expected, since this was the priming batch and manufactured before ASFV was introduced into the feed manufacturing system. For Batch 2 feed samples, ASFV DNA was detected in feed samples across all holding times (Days 0–180). After each successive batch of feed, the quantity of ASFV DNA generally decreased as holding times increased (Table 1). For ASFV P72 genetic material, there was a marginally significant batch × day interaction for Ct value (*P* = 0.072) and a significant batch × day interaction for log_10_ genomic CN/g (*P* = 0.023) in feed samples, indicating the batch of feed and days held at room temperature impacted the quantity of ASFV DNA. Quantity of log_10_ genomic CN/g in feed samples from Batches 2 to 3 did not differ across holding dates (*P* > 0.05). For Batch 4, the quantity of ASFV detected was lower (*P* < 0.05) on Day 7 compared to Day 1 with the other days of analysis being intermediate. In Batch 5, the quantity of ASFV detected was lower (*P* < 0.05) on Day 60 compared to Day 1 with the other days of analysis being intermediate. While in Batch 6, the quantity of ASFV detected was lower (*P* < 0.05) on Days 28 and 180 compared to Day 1 with the other days of analysis being intermediate. For Batch 6, following 180 days storage at room temperature there was no detectable ASFV DNA. However, in Batches 2–5 there was ASFV DNA detected even after 180 days storage. Thus, for the inoculated batch of feed and the three subsequent batches, there was detectable ASFV DNA at the conclusion of the experiment. The only batch of feed that did not contain ASFV DNA was Batch 6 which did not contain detectable genetic material on Days 28 and180 of storage. The variability of ASFV DNA in the feed samples for each batch after different holding times is most likely due to how feed samples were collected for PCR analysis and how each successive batch started with an unknown, diluted, amount of ASFV DNA. In general, these findings are similar to Niederwerder et al. [5] where the detection of ASFV DNA was stable over time in inoculated feed, although that research only evaluated the inoculated feed directly and not subsequent batches of feed through the equipment. While the detection of ASFV DNA is quite stable over time in inoculated feed, the current study indicated that ASFV DNA in later batches of feed was not as stable over time and thus may indicate that holding feed is a potential technique to lessen contamination risk for feed mills to employ.

The main effect of batch (*P* < 0.0001) and day (*P* = 0.0001) were statistically significant for Ct values (Table 2). In Batch 2, the quantity of ASFV DNA detected was greater than Batches 4, 5, and 6 (*P* < 0.05) indicating that the batch that was experimentally inoculated had the greatest amount of ASFV DNA while subsequent noninoculated batches had lower amounts of ASFV DNA. For holding dates, Day 1 feed samples had more ASFV DNA compared to Days 7, 60, and 180 (*P* < 0.05) with all other holding dates intermediate (*P* < 0.05) indicating that feed samples analyzed on the day of feed manufacturing had greater amounts of ASFV DNA detected and that the quantity of ASFV DNA decreased as feed was held for periods of time.

For log_10_ genomic copies/g of feed, main effects of batch (*P* < 0.0001) and day (*P* < 0.0001) were statistically significant. Similar to the Ct values, Batch 2 feed samples had greater quantities of ASFV detected compared to Batches 4, 5, and 6 (*P* < 0.05) indicating that the batch that was experimentally inoculated had the greatest amount of ASFV DNA while subsequent ASFV-free batches had lower amounts of ASFV DNA. For holding dates, Day 1 feed samples had greater amounts of ASFV DNA compared to all other holding dates except for Day 7 which was intermediate (*P* < 0.05) indicating that amount of ASFV DNA was greatest on the day of manufacturing and decreased as feed samples were held for extended periods of time suggesting natural decay of the virus occurred.

When introduced into the feed mill environment after experimental inoculation, ASFV DNA was detected on environmental discs even after long periods of RT storage (Table 3). For ASFV P72 genetic material, there was no evidence of a change in Ct value (*P* = 0.449) or log_10_ genomic CN/mL (*P* = 0.433) of environmental samples from environmental discs over time. Thus, even after 180 days of RT storage, the amount of ASFV DNA detected on environmental surfaces was equal to the amount detected on the day of feed manufacture. These findings are similar to the work by Nuanualsuwan et al. [21] who detected experimentally inoculated ASFV DNA on metal surfaces that were held for extended time at different temperatures. However, it should be noted that [21] evaluated viral persistence for only 7 days and then calculated a model to determine the length of time that ASFV could be detected on various surfaces. To our knowledge, the study reported herein is the first work to document detectable ASFV DNA introduced through contaminated feed in the environmental samples after holding the samples at RT for this length of time. These results indicate that environmental samples could be utilized to detect ASFV DNA contamination within feed mill environments for at least 6 months after initial contamination.

Within this study, the stability of ASFV DNA was evaluated within viral inoculated feed, subsequent batches of feed, as well as on environmental surfaces for 180 days storage at RT. The stability of ASFV DNA in feed during storage differed depending on the batch of feed with the inoculated batch of feed having stable levels of ASFV DNA throughout the storage period, but a reduction was observed over time in the last batch of feed with no detectable ASFV DNA observed following 180 days of storage. Thus, extended holding of feed may reduce the quantity of detectable ASFV DNA, but depending on the level of initial contamination ASFV DNA may still be present up to 180 days at RT storage. Feed is a complex matrix of proteins, carbohydrates, lipids and other components compared to stainless steel surfaces. Under the conditions of this study, the stability of ASFV DNA was greater on environmental surfaces and feed inoculated with high concentrations of ASFV compared to a reduction over time for the later batches of feed where initial concentration of detectable ASFV DNA was lower. These data can be used to help guide future diagnostic investigations and would indicate that ASFV DNA is very stable in a feed mill environment.

A limitation of this experiment is the absence of infectivity data associated with the feed and environmental samples, which is an important part to fully understand risk. However, the focus of this experiment was to evaluate the detection of ASFV DNA which is a rapid and practical method that could be readily employed to screen samples as opposed to virus isolation which requires a BSL-3 facility with special clearances. The data presented provide significant value by establishing the presence of ASFV in feed and the feed mill environment after its introduction through contaminated feed, throughout subsequent batches, and over time at RT storage. This information can be used to help guide epidemiological investigations as the current data shows that ASFV DNA is extremely stable in swine feed and on feed manufacturing surfaces. Thus, if ASFV contamination were present within a feed manufacturing facility, this data have demonstrated the feasibility of detecting also infectious virus in the environment utilizing the simple and convenient sampling methods listed herein.

In conclusion, holding feed for periods of time at RT can decrease ASFV DNA contamination but does not necessarily eliminate ASFV DNA entirely. This study also provides evidence that once ASFV is introduced into the feed mill environment, it will remain in the feed mill for long periods of time (at least 180 days under the conditions used in this study). Fortunately, these data also highlight the fact that ASFV markers can be detected over long periods of time in feed and environmental samples by methods described here. Further research is needed to evaluate potential methods to reduce the ASFV contamination either in feed or the environment that is applicable for the commercial feed mills.

## Figures and Tables

**Table 1:** Proportion of qPCR positive and interactive means of cycle threshold (Ct) value and log_10_ genomic copies/g of feed samples for ASFV DNA survival after experimental inoculation of swine feed and subsequent feed batch sequencing.

Item	Batch of feed^†^				
	2	3	4	5	6
Proportion PCR positive					
Day 1	40/40	20/20	19/20	19/20	17/20
Day 3	6/6	4/6	3/6	3/6	1/6
Day 7	6/6	5/6	1/6	2/6	2/6
Day 14	6/6	6/6	4/6	5/6	3/6
Day 28	6/6	6/6	3/6	3/6	0/6
Day 60	6/6	6/6	3/6	1/6	2/6
Day 90	6/6	6/6	2/6	2/6	1/6
Day 180	6/6	5/6	2/6	2/6	0/6
Cycle threshold^‡^					
Day 1	33.0^a^	37.5^b,c,d,e^	39.5^e,f,g,h,i^	39.3^e,f,g,h,i^	40.1^e,f,g,h,i^
Day 3	31.7^a,b^	39.5^c,d,e,f,g,h,i^	42.4^e,f,g,h,i^	41.4^e,f,g,h,i^	43.8^g,h,i^
Day 7	31.6^a^	37.8^a,b,c,d,e,f,g,h^	44.3^h,i^	42.2^e,f,g,h,i^	43.5^f,g,h,i^
Day 14	31.8^a,b^	36.9^a,b,c,d,e,f,g^	40.6^e,f,g,h,i^	39.6^d,e,f,g,h,i^	41.4^e,f,g,h,i^
Day 28	31.3^a^	36.5^a,b,c,d,e,f^	42.7^e,f,g,h,i^	42.5^e,f,g,h,i^	45.0^i^
Day 60	32.4^a,b,c^	37.8^a,b,c,d,e,f,g,h^	42.3^e,f,g,h,i^	44.3^h,i^	43.2^e,f,g,h,i^
Day 90	32.6^a,b,c,d^	36.2^a,b,c,d,e^	43.0^e,f,g,h,i^	43.6^f,g,h,i^	43.6^f,g,h,i^
Day 180	32.0^a,b^	39.7^d,e,f,g,h,i^	43.5^f,g,h,i^	41.5^e,f,g,h,i^	45.0^i^
Log_10_ genomic copies/g^§^					
Day 1	4.7^i^	3.6^f,g,h,i^	3.1^d,e,f,g,h^	3.1^c,d,e,f,g,h^	2.8^b,c,d,e,f,g,h^
Day 3	5.0^h,i^	2.5^a,b,c,d,e,f,g,h,i^	1.5^a,b,c,d,e,f^	1.7^a,b,c,d,e,f,g^	0.6^a,b,c^
Day 7	5.0^h,i^	3.2^b,c,d,e,f,g,h,i^	0.5^a,b^	1.3^a,b,c,d,e,f^	0.9^a,b,c,d,e^
Day 14	4.9^h,i^	3.7^b,c,d,e,f,g,h,i^	2.2^a,b,c,d,e,f,g,h^	2.7^a,b,c,d,e,f,g,h,i^	1.7^a,b,c,d,e,f,g^
Day 28	5.1^h,i^	3.8^b,c,d,e,f,g,h,i^	1.4^a,b,c,d,e,f^	1.5^a,b,c,d,e,f^	0.00^a^
Day 60	4.8^g,h,i^	3.5^e,f,g,h,i^	1.5^a,b,c,d,e,f^	0.5^a,b^	1.0^a,b,c,d,e^
Day 90	4.7^g,h,i^	3.8^b,c,d,e,f,g,h,i^	1.1^a,b,c,d,e^	0.9^a,b,c,d,e^	0.6^a,b,c,d^
Day 180	4.9^h,i^	2.7^e,f,g,h,i^	0.9^a,b,c,d,e^	1.4^a,b,c,d,e,f^	0.0^a^

*Note*: ^†^Swine gestation feed was inoculated with African swine fever virus (ASFV) for a final concentration of 5.6 × 104 TCID50/gram inoculated feed (Batch 2) following an initial priming of the feed manufacturing equipment with ASFV free feed. Four subsequent batches of initially ASFV-free feed were then manufactured (Batches 3–6). On Days 1, 3, 7, 14, 28, 60, 90, and 180 after manufacture following room temperature storage, three samples were mixed with approximately 35 mL of phosphate buffered solution, incubated for 2 hr at room temperature then centrifuged at 1,000 × *g* for 3 min. Samples were then analyzed using qRT-PCR for detection of the gene encoding for the p72 protein. Analysis of Day 1 feed samples have been reported by Elijah et al. [12] and are included in the current analysis of ASFV detection over time. ^‡^Samples that had no detectable ASFV DNA were assigned a Ct value of 45.0. Batch × day: *P* = 0.072. SEM for Batch 2, Day 1 = 0.64; SEM for Batches 3–6, Day 1 = 0.69; All other SEM = 1.27. ^§^Log_10_ genomic copies/g of feed. Batch × day, *P* = 0.023. SEM for Batch 2, Day 1 = 0.27; SEM for Batches 3–6, Day 1 = 0.30; All other SEM = 0.56. ^a–i^Means within item lacking common superscript differ (*P* < 0.05) using Tukey multiple comparison adjustment.

**Table 2:** Proportion of qPCR positive and main effects of batch and day on cycle threshold (Ct) value and log_10_ genomic copies/g for feed held in room temperature storage for ASFV DNA survival after experimental inoculation of swine feed and subsequent feed batch sequencing.

Item^†^	Proportion PCR positive	Cycle threshold value^‡^	Log_10_ genomic copies/g^§^
Batches of feed			
2	82/82	32.1^a^	4.9^c^
3	58/62	37.7^b^	3.3^b^
4	37/62	41.8^c^	1.5^a^
5	37/62	42.3^c^	1.6^a^
6	26/62	43.2^c^	1.0^a^
Day			
1	115/120	37.9^a^	3.5^b^
3	17/30	39.8^a,b^	2.2^a^
7	16/30	39.9^b^	2.2^a^
14	24/30	38.1^a,b^	3.0^a,b^
28	18/30	39.6^a,b^	2.3^a^
60	18/30	40.0^b^	2.2^a^
90	17/30	39.8^a,b^	2.2^a^
180	15/30	40.3^b^	2.0^a^

*Note*: ^†^Swine gestation feed was inoculated with African swine fever virus (ASFV) for a final concentration of 5.6 × 104 TCID50/gram (Batch 2) following an initial priming of the feed manufacturing equipment with ASFV free feed. Four subsequent batches of initially ASFV-free feed were then manufactured (Batches 3–6). On Day 1, 3, 7, 14, 28, 60, 90, and 180 after manufacture following room temperature storage, three samples were mixed with approximately 35 mL of phosphate buffered solution, incubated for 2 hr at room temperature then centrifuged at 1,000 × *g* for 3 min. Samples were then analyzed using qRT-PCR for detection of the gene encoding for the p72 protein. Analysis of Day 1 feed samples have been reported by Elijah et al. [12] and are included in the current analysis of ASFV detection over time. ^‡^Samples that had no detectable ASFV DNA were assigned a Ct value of 45.0. Batch, *P* < 0.0001, SEM = 0.43; Day, SEM for Day 1 = 0.31, otherwise SEM = 0.57. ^§^Log_10_ genomic copies/g feed. Batch, *P* < 0.0001, SEM = 0.19; Day, *P* < 0.0001, SEM for Day 1 = 0.13; otherwise SEM = 0.25. ^a–c^Means within item lacking common superscript differ (*P* < 0.05) using Tukey multiple comparison adjustment.

**Table 3:** Proportion of qPCR positive and main effects of location on cycle threshold (Ct) and log_10_ genomic copies/mL of environmental discs for ASFV DNA survival after experimental inoculation of swine feed and subsequent feed batch sequencing.

Day^†^	Proportion PCR positive	Cycle threshold value^‡^	Log_10_ genomic copies/mL^§^
1	6/6	33.8	3.9
3	6/6	34.0	3.9
7	6/6	35.3	3.6
14	5/6	36.7	3.0
28	6/6	33.9	3.9
60	6/6	37.7	2.9
90	6/6	35.5	3.5
180	4/6	39.3	2.2

*Note*: ^†^Twenty-seven stainless steel coupons were randomly placed in location (nine coupons in each of three locations of the room) and allowed to collect feed dust produced during manufacture. Stainless steel coupons remained sealed in a secondary container and stored at room temperature (RT) in a locked cabinet. On Day of and 3, 7, 14, 28, 60, 90, and 180 days after feed manufacturing, one sample from each of the three location blocks following RT storage were randomly selected, opened within a BSC, swabbed using a 10 × 10 cm cotton gauze, prepared and analyzed as for ASFV DNA via PCR. ^‡^Samples that had no detectable ASFV DNA were assigned a Ct value of 45.0. Day: *P* = 0.449, SEM = 1.98. ^§^Genomic copies/mL of sample processing lysate. Day: *P* = 0.433, SEM = 0.60.

## Data Availability

Data may be provided by the corresponding author upon reasonable request.

## References

[1] Niederwerder M. C. (2021). Risk and mitigation of African swine fever virus in feed. *Animals*.

[2] Dee S. A., Bauermann F. V., Niederwerder M. C. (2018). Survival of viral pathogens in animal feed ingredients under transboundary shipping models. *PLoS ONE*.

[3] Stoian A. M. M., Petrovan V., Constance L. A. (2020). Stability of classical swine fever virus and pseudorabies virus in animal feed ingredients exposed to transpacific shipping conditions. *Transboundary and Emerging Diseases*.

[4] Stoian A. M. M., Zimmerman J., Ji J. (2019). Half-life of African swine fever virus in shipped feed. *Emerging Infectious Diseases*.

[5] Niederwerder M. C., Khanal P., Foland T. (2022). Stability of African swine fever virus in feed during environmental storage. *Transboundary and Emerging Diseases*.

[6] Petrini S., Feliziani F., Casciari C., Giammarioli M., Torresi C., De Mia G. M. (2019). Survival of African swine fever virus (ASFV) in various traditional Italian dry-cured meat products. *Preventative Veterinary Medicine*.

[7] McKercher P. D., Yedloutschnig R. J., Callis J. J. (1987). Survival of viruses in, “Prosciutto di Parma”, (Parma Ham). *Canadian Institute of Food Science and Technology Journal*.

[8] Zani L., Masiulis M., Bušauskas P. (2020). African swine fever virus survival in buried wild boar carcasses. *Transboundary and Emerging Diseases*.

[9] Davies K., Goatley L. C., Guinat C. (2017). Survival of African swine fever virus in excretions from pigs experimentally infected with the Georgia 2007/1 isolate. *Transboundary and Emerging Diseases*.

[10] Houston G. E. (2023). *The emergence, distribution, and persistence of viral pathogens in swine production systems*.

[11] Elijah C. G., Trujillo J. D., Jones C. K. (2021). Evaluating the distribution of African swine fever virus within a feed mill environment following manufacture of inoculated feed. *PLoS ONE*.

[12] Elijah C. G., Trujillo J. D., Jones C. K. (2022). Effect of mixing and feed batch sequencing on the prevalence and distribution of African swine fever virus in swine feed. *Transboundary and Emerging Diseases*.

[13] Jones C., Stewart S., Woodworth J., Dritz S., Paulk C. (2020). Validation of sampling methods in bulk feed ingredients for detection of swine viruses. *Transboundary and Emerging Diseases*.

[14] Li Z., Chen W., Qiu Z. (2022). African swine fever virus: a review. *Life*.

[15] Blome S., Franzke K., Beer M. (2020). African swine fever—a review of current knowledge. *Virus Research*.

[16] Gebhardt J. T., Dritz S. S., Elijah C. G., Jones C. K., Paulk C. B., Woodworth J. C. (2022). Sampling and detection of African swine fever virus within a feed manufacturing and swine production system. *Transboundary and Emerging Diseases*.

[17] Shurson G. C., Urriola P. E. (2019). *Understanding the vitamin supply chain and relative risk of transmission of foreign animal diseases*.

[18] Paulino-Ramirez R., Jimenez J. A. (2021). Food security and research agenda in African swine fever virus: a new arbovirus threat in the Dominican Republic. *InterAmerican Journal of Medicine and Health*.

[19] Huss A. R., Schumacher L. L., Cochrane R. A. (2017). Elimination of porcine epidemic diarrhea virus in an animal feed manufacturing facility. *PLoS ONE*.

[20] Schumacher L. L., Cochrane R. A., Huss A. R. (2018). Feed batch sequencing to decrease the risk of porcine epidemic diarrhea virus (PEDV) cross-contamination during feed manufacturing. *Journal of Animal Science*.

[21] Nuanualsuwan S., Songkasupa T., Boonpornprasert P., Suwankitwat N., Lohlamoh W., Nuengjamnong C. (2022). Persistence of African swine fever virus on porous and non-porous fomites at environmental temperatures. *Porcine Health Management*.

